# Low or undetectable TPO receptor expression in malignant tissue and cell lines derived from breast, lung, and ovarian tumors

**DOI:** 10.1186/1471-2407-12-405

**Published:** 2012-09-11

**Authors:** Connie L Erickson-Miller, Kodandaram Pillarisetti, Jennifer Kirchner, David J Figueroa, Lone Ottesen, Anne-Marie Martin, Yuan Liu, Yasser Mostafa Kamel, Conrad Messam

**Affiliations:** 1 GlaxoSmithKline, 1250 South Collegeville Rd, Collegeville, PA, 19426, USA; 2 GlaxoSmithKline, Stockley Park, Uxbridge, Middlesex UB11 1BT, UK

## Abstract

**Background:**

Numerous efficacious chemotherapy regimens may cause thrombocytopenia. Thrombopoietin receptor (TPO-R) agonists, such as eltrombopag, represent a novel approach for the treatment of chemotherapy-induced thrombocytopenia. The TPO-R *MPL* is expressed on megakaryocytes and megakaryocyte precursors, although little is known about its expression on other tissues.

**Methods:**

Breast, lung, and ovarian tumor samples were analyzed for *MPL* expression by microarray and/or quantitative reverse transcription-polymerase chain reaction (qRT-PCR), and for TPO-R protein expression by immunohistochemistry (IHC). Cell line proliferation assays were used to analyze the in vitro effect of eltrombopag on breast, lung, and ovarian tumor cell proliferation. The lung carcinoma cell lines were also analyzed for TPO-R protein expression by Western blot.

**Results:**

*MPL* mRNA was not detectable in 118 breast tumors and was detectable at only very low levels in 48% of 29 lung tumors studied by microarray analysis. By qRT-PCR, low but detectable levels of *MPL* mRNA were detectable in some normal (14-43%) and malignant (3-17%) breast, lung, and ovarian tissues. A comparison of *MPL* to *EPOR*, *ERBB2,* and *IGF1R* mRNA demonstrates that *MPL* mRNA levels were far lower than those of *EPOR* and *ERBB2* mRNA in the same tissues. IHC analysis showed negligible TPO-R protein expression in tumor tissues, confirming mRNA analysis. Culture of breast, lung, and ovarian carcinoma cell lines showed no increase, and in fact, showed a decrease in proliferation following incubation with eltrombopag. Western blot analyses revealed no detectable TPO-R protein expression in the lung carcinoma cell lines.

**Conclusions:**

Multiple analyses of breast, lung, and ovarian tumor samples and/or cell lines show no evidence of *MPL* mRNA or TPO-R protein expression. Eltrombopag does not stimulate growth of breast, lung, or ovarian tumor cell lines at doses likely to exert their actions on megakaryocytes and megakaryocyte precursors.

## Background

Breast cancer is the most commonly occurring neoplasm and the second leading cause of cancer deaths in women. Lung cancer is the second most frequent cancer diagnosis in men and women, and remains the leading cause of cancer deaths. Although ovarian cancer affects fewer women than breast or lung cancer, it is one of the most lethal types of cancer [1]. Current clinical guidelines recommend platinum-containing chemotherapy regimens, among others, for these malignancies in different disease stages [2-7]. Established treatment protocols may be associated with a range of adverse events (AEs), including thrombocytopenia, anemia, and neutropenia. Newer chemotherapy combinations including carboplatin plus pemetrexed or gemcitabine in non-small cell lung cancer (NSCLC) may also be associated with high rates of thrombocytopenia [8].

Thrombocytopenia may lead to significant clinical consequences including petechiae, gastrointestinal bleeding, and bleeding into the brain [9]. Neutropenia, anemia, or thrombocytopenia resulting from bone marrow suppression can delay chemotherapy administration and/or may prompt dose reductions, with possible negative impact on disease control [10]. The use of hematopoietic growth factors has ameliorated this problem to some degree with respect to red and white blood cell production [11,12]. However, there are concerns that some growth factors could induce proliferation of other cell types, including tumor cells [13,14].

Thrombopoietin (TPO) is a critical cytokine regulating thrombopoiesis. It is the endogenous ligand for the thrombopoietin receptor (TPO-R) *MPL* expressed on the surface of megakaryocytes, megakaryocyte precursors, and platelets [15-18]. TPO-R agonists are approved for the treatment of chronic immune thrombocytopenia (ITP). The interaction of the selective, nonpeptidyl TPO-R agonist, eltrombopag, with TPO-R triggers activation of the JAK-STAT and MAP kinase, but not the AKT, signal transduction pathways. This causes alterations in gene expression patterns to promote megakaryocytic differentiation and maturation, resulting in increased platelet counts [19,20]. There are differences between the amplitude and extent of signaling between TPO and eltrombopag [21]. Acute myeloid leukemia (AML) blasts have been shown to express TPO-R [22] and in some reports TPO has induced proliferation of these blasts [23], although investigations in leukemia cell lines show conflicting results [24,25]. A variety of human non-megakaryocytic leukemia and lymphoma cell lines show decreased, rather than increased, proliferation upon incubation with eltrombopag [26]. In vitro and in vivo analyses of bone marrow mononuclear cells from patients with AML and myelodysplastic syndromes (MDS) similarly showed no increase in proliferation with eltrombopag treatment [25,27].

The objective of this study was to determine whether solid tumors (i.e., breast, lung, and ovarian) express *MPL* mRNA or TPO-R protein, and whether eltrombopag affects the proliferation of solid tumor cell lines. Expression of *MPL* mRNA was assessed by microarray analysis in breast and lung tumor samples and by quantitative reverse transcription-polymerase chain reaction (qRT-PCR) in normal and malignant tissue samples from breast, lung, and ovary. TPO-R protein expression was determined by immunohistochemistry (IHC) on breast, lung, and ovarian tumor samples and by western blot on lung cell lines. Cell proliferation in response to eltrombopag was evaluated in breast, lung, and ovarian cancer cell lines.

## Methods

### Patient samples

In accordance with the Helsinki Declaration, all patients provided written informed consent for use of their samples, and the collection and use of the samples received Institutional Review Board (IRB) approval.

### Microarray analysis

#### Experimental samples

Archival tissue samples from 118 patients who had locally advanced or metastatic breast cancer and had failed treatment with anthracycline-, taxane-, and trastuzumab-containing regimens were studied. Specimens were obtained from patients who had histologically confirmed invasive breast cancer with Stage IIIB, Stage IIIC with T4 lesion, or Stage IV disease (GSK Study EGF100151), with documentation of *ERBB2* overexpression (IHC 3+ or IHC 2+ with fluorescence in situ hybridization [FISH] confirmation) [28,29]. Microarray analysis was performed at Response Genetics, Inc (Los Angeles, CA, USA).

Frozen tissue samples from 29 patients who were presurgical, treatment-naive, with Stage IA or Stage IB, resectable NSCLC were studied (GSK Study VEG105290). Microarray analysis was performed at Weill Medical College of Cornell University (New York, NY, USA).

#### Experimental protocol

Archival breast tumor specimens were formalin-fixed and embedded in paraffin. The quality of tumor tissue RNA extraction was assessed by laser capture microdissection. Reverse transcription-polymerase chain reaction (RT-PCR) for 300 base pair (bp) of the β-actin gene was used for quality control of these samples. Samples with a cycle time (CT) of < 32 at the 300 bp level were acceptable for microarray analysis. mRNA and cDNA were prepared from archival breast tumor tissues and frozen NSCLC samples using the Affymetrix HG-U133 Plus2 array (Affymetrix, Inc., Santa Clara, CA, USA). RNA microarray gene signal intensity was normalized using the robust microchip analysis (RMA) method described by Irizarry et al [30]. Samples with RMA < 50 are below the level of detection by this method.

### qRT-PCR analysis

#### Experimental samples

Samples of normal tissue cDNA and tumor tissue cDNA were obtained from Cytomyx (Lexington, MA, USA). Seven samples of normal breast tissue were studied, and 41 breast carcinoma samples, including tissue from cancers of Stage I, Stage II, and Stage IIIB were analyzed. Eight normal lung specimens and lung tissue samples from 40 tumors, ranging from Stages IA to IV were examined. Ovarian samples included 7 normal ovarian tissues and 41 ovarian tumors ranging from Stages I to IV.

#### Experimental protocol

*MPL* gene expression was measured using FAM-TAMRA-labeled primers and probes in a 7900HT thermal cycler using a standard 40-cycle profile with 9600 emulation. Following amplification, calculations were performed to determine the relative abundance of *MPL* normalized to 3 housekeeping genes, glyceraldehyde-3-phosphate dehydrogenase (*GAPDH*), β-actin (*ACTB)*, and cyclophilin A (*PPIA*). Each result is presented as a normalized value of mRNA. *EPOR*, *ERBB2,* and *IGF1R* were also assessed to provide data for comparable receptors. Primers and probes were custom made by Integrated DNA Technologies (Coralville, IA, USA). Sequences for the primers and probes were previously described [31] and are listed in Additional file 1: Table S1.

#### qRT-PCR data analysis

CT values for genes of interest were normalized to the internal housekeeping genes run in the reaction using in-house software. Raw abundance value was calculated using the following equation: Abundance = 10e[(40-CT)/3.35]. Samples were scaled relative to each other using the geometric mean of the set of valid housekeeping gene data points for that sample. Each data point was then expressed as the ratio of the housekeeping gene abundance in the sample to the average of that housekeeping gene in all samples and marked invalid if it had statistically inconsistent behavior with the other housekeeping genes in those samples of similar tissue types. Samples with a relative abundance of < 50 are below the level of detection of this method.

### IHC for TPO-R protein expression

#### Experimental samples

Formalin-fixed, paraffin embedded (FFPE) controls and FFPE specimens of breast, lung, and ovarian cancer were procured by Mosaic Laboratories under an IRB-reviewed protocol that allows for use of remnant, de-identified, or anonymized human samples for in vitro analysis under the guidelines defining Exemption from Human Subject Research as defined by the Office of Human Research Protection. FFPE tissue samples of breast, lung, and ovarian cancer were also procured from OriGene (formerly Cytomyx) for analysis. A FFPE block of N2C-Tpo cells, a Tpo-dependent megakaryocytic leukemia cell line, and of normal bone marrow were used as positive controls for TPO-R expression.

#### Experimental protocol

Immunohistochemical analysis was performed in accordance with Mosaic Laboratories’ Standard Operating Procedures. Briefly, the procedure was performed manually using the Envision™ system and ancillary reagents (Dako, Carpinteria, CA). Specimens were sectioned at a thickness of 4 microns, mounted onto positive-charged glass slides, dried, deparaffinized, and rehydrated. Following rehydration, tissue sections were incubated in Envision peroxidase for 5 minutes to quench endogenous peroxidase. Heat-induced epitope retrieval was performed using Rip Tide buffer for 40 minutes at 95°C. Slides were incubated with anti-CD110 antibody *(clone 1.78.1)* (BD Biosciences) diluted in diluent (Dako) for 30 minutes. Slides were then rinsed twice in Splash-T buffer for 5 minutes each. Signal was visualized using Envision + Mouse HRP detection reagent (Dako) for 30 minutes, followed by 3,3 diamino benzidine according to manufacturer’s instructions. Slides were rinsed with water, counterstained with hematoxylin (Dako), dehydrated through graded alcohols, cleared in xylene, and coverslipped for microscopic evaluation.

#### Data analysis

Staining was evaluated by a pathologist; evaluation of reactivity involved a combination of the following: cellular localization of staining, staining intensity, subcellular localization, and percentage of cells staining in the primary component of the tissue type of interest. The CD110 IHC assay was scored on a semi-quantitative scale, and the percentage of cancer cells staining at each of the following 4 levels was recorded: 0 (unstained), 1+ (weak staining), 2+ (moderate staining), and 3+ (strong staining). An H-score was calculated based on the summation of the product of percent of cells stained at each intensity, using the following equation: (3 × % cells staining at 3+) + (2 × % cells staining at 2+) + (1 × % cells staining at 1+). H-score values range from 0–300. (Additional file 2: Table S2).

### Cell line proliferation assays

#### Experimental preparations

Cell lines were obtained from the American Type Culture Collection (Walkersville, MD, USA). All cells were maintained in log-phase growth in their respective media. The breast cancer cell lines (MCF-7, BT474, and HCC1937), lung cancer cell lines (A549, NCI-H226, NCI-H460, and NCI-H510), and ovarian cell lines (OVCAR4 and SKOV-3) were grown in RPMI 1640 medium with 10% fetal calf serum (FCS). The ovarian carcinoma cell line OVCAR3 was grown in RPMI 1640 medium with 20% FCS, 1% sodium pyruvate, and 1% v/v glutamine.

All experiments were performed with milled, monoethanolamine salt form SB-497115-GR (eltrombopag) resuspended in water to 10 mg/mL, and diluted in Iscove’s Modified Dulbecco’s Medium (IMDM) with 1% FCS. Recombinant human TPO (rhTPO) was purchased from R&D Systems (Minneapolis, MN, USA) and diluted in IMDM.

#### Experimental protocols

Cells for the Cell Titer Glo® assay were plated at 1 × 10^3^ cells/well in 96-well plates in medium containing 10% FCS and allowed to adhere for 24 hours. Cells were treated with eltrombopag at 0, 0.1, 0.4, 1, 4, 10, and 40 μg/mL. In breast and ovarian cancer cell lines, eltrombopag was also tested at 100 μg/mL. rhTPO at 100 ng/mL was also tested in these experiments. Cells were incubated for 72 hours at 37°C in 5% CO_2_ after the addition of eltrombopag or rhTPO. Active cell determinations were performed using the Cell Titer Glo® reagent (Promega, Madison, WI, USA) according to the manufacturer’s protocol. Results were reported as relative luminescence units (RLU).

#### Data analysis

The calculated mean and standard deviations were produced using triplicate samples for each experiment. The IC_50_ was determined using XLfit version 4.2.1, utilizing the best fit model for each data set.

### Western blotting for TPO-R protein expression

#### Experimental preparations

Lung cancer cell lines, A549, NCI-H226, NCI-H460, and NCI-H510, were grown as described above.

#### Experimental protocol

Western blots for TPO-R protein expression were performed on reduced cell lysates of log-phase growth lung cancer cell lines (50 μg/well) on a NuPage 4-12% Bis-Tris gel (Invitrogen, Carlsbad, CA, USA) with MOPS running buffer. Precision Protein Dual Color Standards (Bio-Rad, Hercules, CA, USA) were used. The gels were transferred to nitrocellulose and stained with a rabbit polyclonal anti-TPO-R primary antibody (Upstate Biotechnology Inc., Lake Placid, NY, USA; Cat# 06–944) and analyzed with an Odyssey® infrared imager (LI-COR Biosciences, Lincoln, NE, USA).

## Results and discussion

### MPL expression by microarray analysis

**Figure 1:**
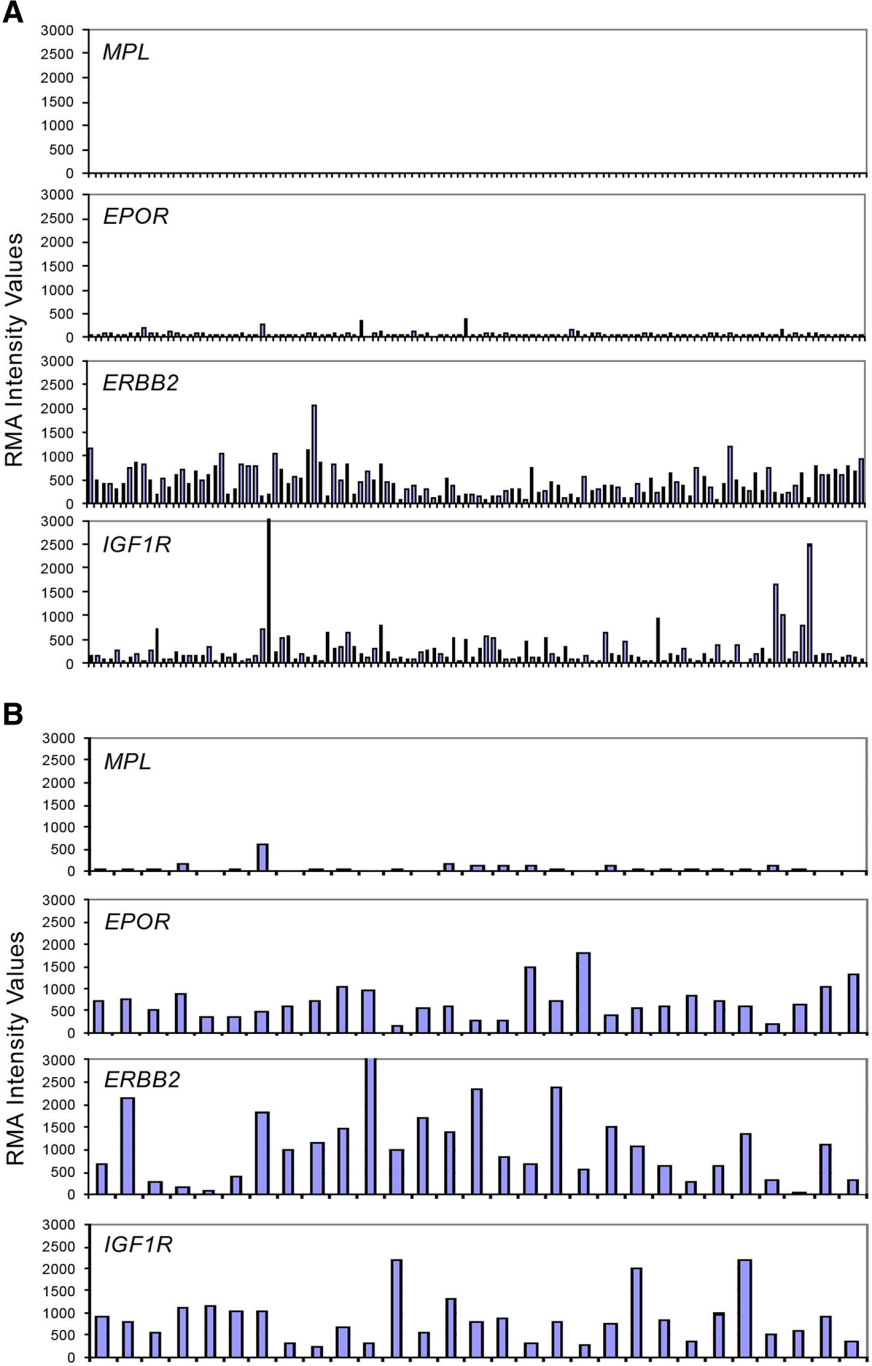
**Expression of *MPL *mRNA message determined by microarray analysis, compared to *EPOR*, *ERBB2, *and *IGF1R *message levels in (A) 118 breast cancer tumor samples (Study EGF100151) and (B) 29 non-small cell lung carcinoma tumor samples (Study VEG105290).** (**A**). Breast cancer. (**B**). Non-small cell lung carcinoma.

None of the 118 advanced or metastatic breast cancer samples expressed detectable levels of *MPL* mRNA; all samples had RMA < 50. *EPOR* mRNA was expressed at detectable levels in 89/118 (75%); all 118 (100%) demonstrated detectable levels of *ERBB2* mRNA; and 102/118 (86%) expressed detectable levels of *IGF1R* mRNA (Table 1, Figure 1). *ERBB2* mRNA was expressed at the highest level compared with *MPL*, *EPOR*, and *IGF1R* mRNA levels.

**Table 1:** **Expression of detectable levels of *MPL*, *EPOR*, *ERBB2*, and *IGF1R *mRNA in primary tumors of breast and NSCLC origin by microarray analysis**

	**Breast N = 118 n (%)**	**NSCLC N = 29 n (%)**
*MPL*	0 (0)	14 (48)
*EPOR*	89 (75)	29 (100)
*ERBB2*	118 (100)	28 (97)
*IGF1R*	102 (86)	29 (100)

NSCLC, non-small cell lung cancer. Data are represented as number of tumors with detectable (RMA ≥ 50) expression and percent of tumor type with detectable expression in parenthesis.

Of the 29 samples of NSCLC studied by microarray, 14 (48%) expressed low but detectable levels of *MPL* mRNA (Table 1, Figure 1). All 29 samples (100%) expressed *EPOR* mRNA; 28 of 29 (97%) expressed *ERBB2* mRNA, and all 29 (100%) expressed *IGF1R* mRNA.

### MPL expression by qRT-PCR analysis

**Figure 2:**
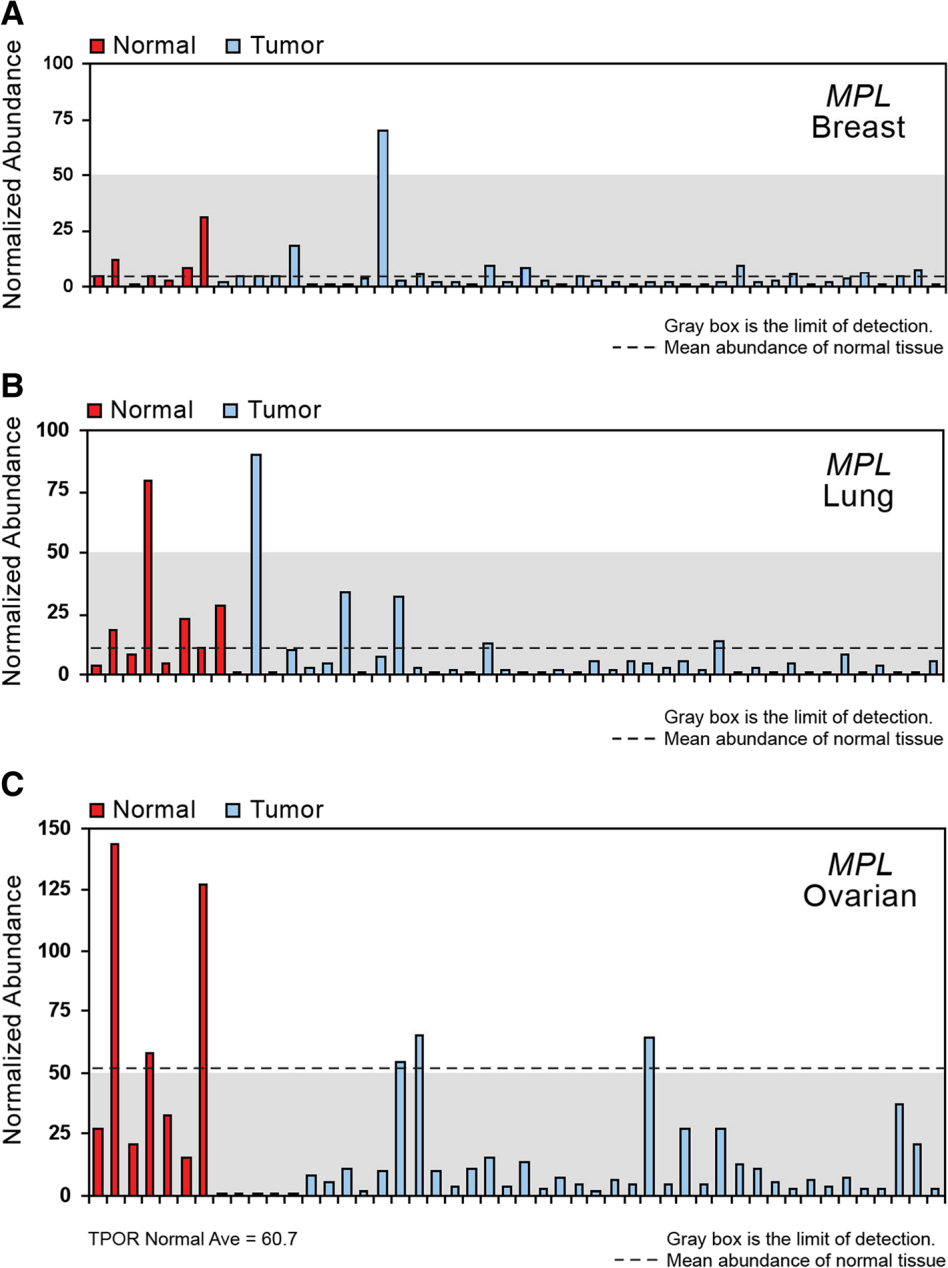
**Relative abundance of *MPL *mRNA expression determined by qRT-PCR in normal (red bars) and tumor (blue bars) samples.** Abundance is normalized to *GAPDH*, β-actin, and cyclophilin. The dashed line represents the mean of the normal samples. The gray box represents relative abundance below the level of accurate detection (abundance < 50). (**A**). Breast. (**B**). Lung. (**C**). Ovarian.

**Figure 3:**
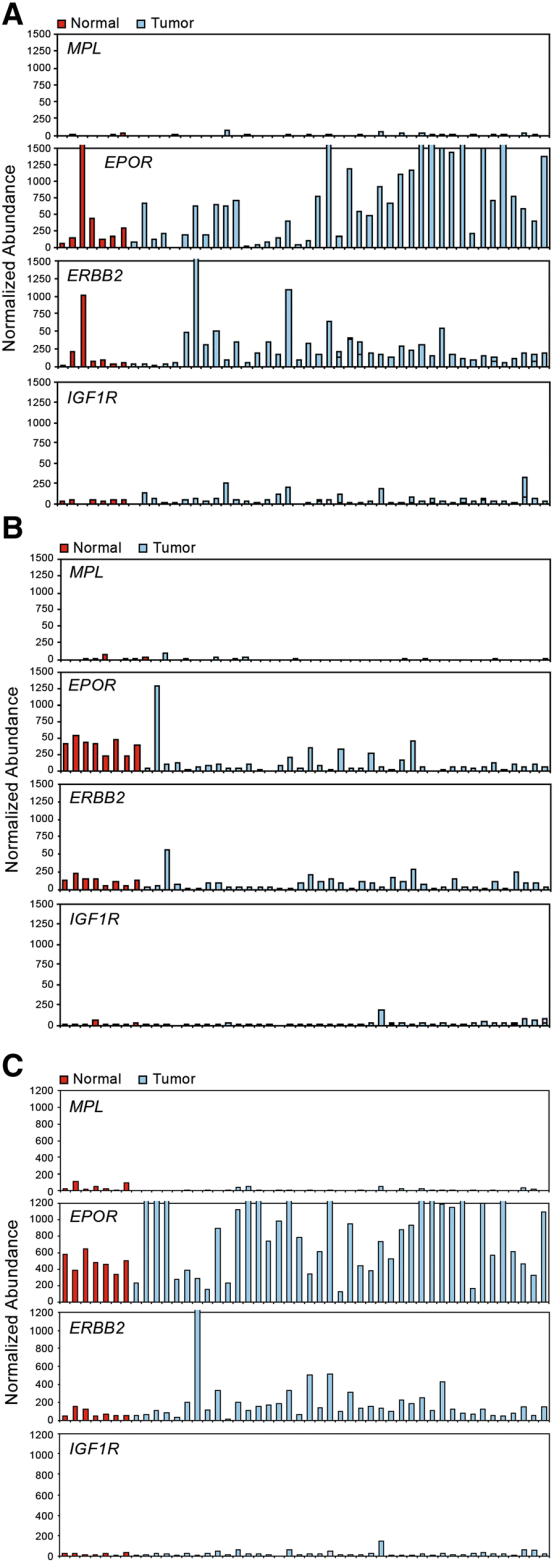
**Relative abundance of *MPL*, *EPOR*, *ERBB2, *and *IGF1R *mRNA expression determined by qRT-PCR in normal and tumor samples.** Abundance is normalized to *GAPDH*, β-actin, and cyclophilin. Red bars are normal samples, blue bars are tumor samples. Relative abundance below 50 is below the level of detection. (**A**). Breast tumor taqman. (**B**). Lung tumor taqman. (**C**). Ovarian tumor taqman.

A more accurate quantitation of *MPL* expression was undertaken using qRT-PCR. *MPL* expression was lower in both normal and malignant breast tissue than expression of *EPOR*, *ERBB2*, and *IGF1R* (Table 2, Figures 2A and 3A). One of 7 (14%) normal breast tissue samples studied and 1/40 (3%) breast tumor samples demonstrated detectable *MPL* mRNA expression (Table 2, Figures 2A and 3A). *EPOR* mRNA expression was observed in 7/7 (100%) normal breast samples, and in 32/40 (80%) breast cancer samples. Four of 7 (57%) normal breast tissue samples and 33/40 (83%) breast tumor samples demonstrated detectable *ERBB2* expression. *IGF1R* expression was detected in 2/7 (29%) normal breast tissue samples and in 15/40 (38%) breast cancer samples.

**Table 2:** **Expression of detectable levels (abundance ≥ 50) of *MPL*, *EPOR*, *ERBB2, *and *IGF1R *mRNA in normal tissue and tumor samples by qRT-PCR**

	**Breast normal N = 7 n (%)**	**Breast tumor N = 40 n (%)**	**Lung normal N = 8 n (%)**	**Lung tumor N = 40 n (%)**	**Ovarian normal N = 7 n (%)**	**Ovarian tumor N = 41 n (%)**
*MPL*	1 (14)	1 (3)	1 (13)	1 (3)	3 (43)	3 (7)
*EPOR*	7 (100)	32 (80)	8 (100)	28 (70)	7 (100)	41 (100)
*ERBB2*	4 (57)	33 (83)	8 (100)	22 (55)	7 (100)	39 (95)
*IGF1R*	2 (29)	15 (38)	1 (13)	2 (5)	0 (0)	7 (17)

Quantitative reverse transcription-polymerase chain reaction was used to measure abundance of these genes. Data are represented as number of samples with detectable expression and percent of the samples in parenthesis.

Detectable *MPL* expression was observed using qRT-PCR in 1/8 (13%) normal lung tissue samples, and in 1/40 (3%) lung tumor samples. The level of *MPL* expression was equivalent in the normal lung tissue and in the positive tumor sample (Table 2, Figures 2B and 3B). The lung tumor sample that expressed detectable *MPL* mRNA also expressed *EPOR* mRNA at levels greater than did other lung tumor samples. *EPOR* mRNA expression was identified in all 8 (100%) normal lung samples, and in 28/40 (70%) lung tumor samples studied. *ERBB2* mRNA expression was detected in all 8 (100%) normal samples and in 22/40 (55%) lung tumor samples. In contrast, *IGF1R* mRNA expression was identified at detectable levels in only 1/8 (13%) normal lung samples and 2/40 (5%) lung tumor samples studied (Table 2, Figures 2B and 3B).

Three of 7 (43%) normal ovarian tissue samples expressed detectable *MPL* mRNA and 3/41 (7 %) ovarian tumor samples had detectable *MPL* mRNA. Relative expression of *MPL* was similar or greater in the positive normal ovary and ovarian tumor samples (Table 2, Figures 2C and 3C). All 7 (100%) normal ovary samples and all 41 (100%) ovarian tumors studied expressed *EPOR* mRNA. *ERBB2* expression was also observed in all 7 (100%) normal samples and in 39/41 (95%) tumor samples. *IGF1R* expression was not detected in the 7 normal ovarian samples, and was detectable in 7/41 (17%) ovarian tumors studied. Expression of *MPL* and *IGF1R* mRNA was low compared with *EPOR* and *ERBB2* mRNA levels in normal ovary and ovarian tumor samples (Table 2, Figures 2C and 3C). There was too little *MPL* mRNA expression in the tumor samples to determine any relationship between *MPL* expression levels and stage of cancer in these breast, lung, or ovarian samples (Figure 3).

### TPO-R protein expression by IHC

**Figure 4:**
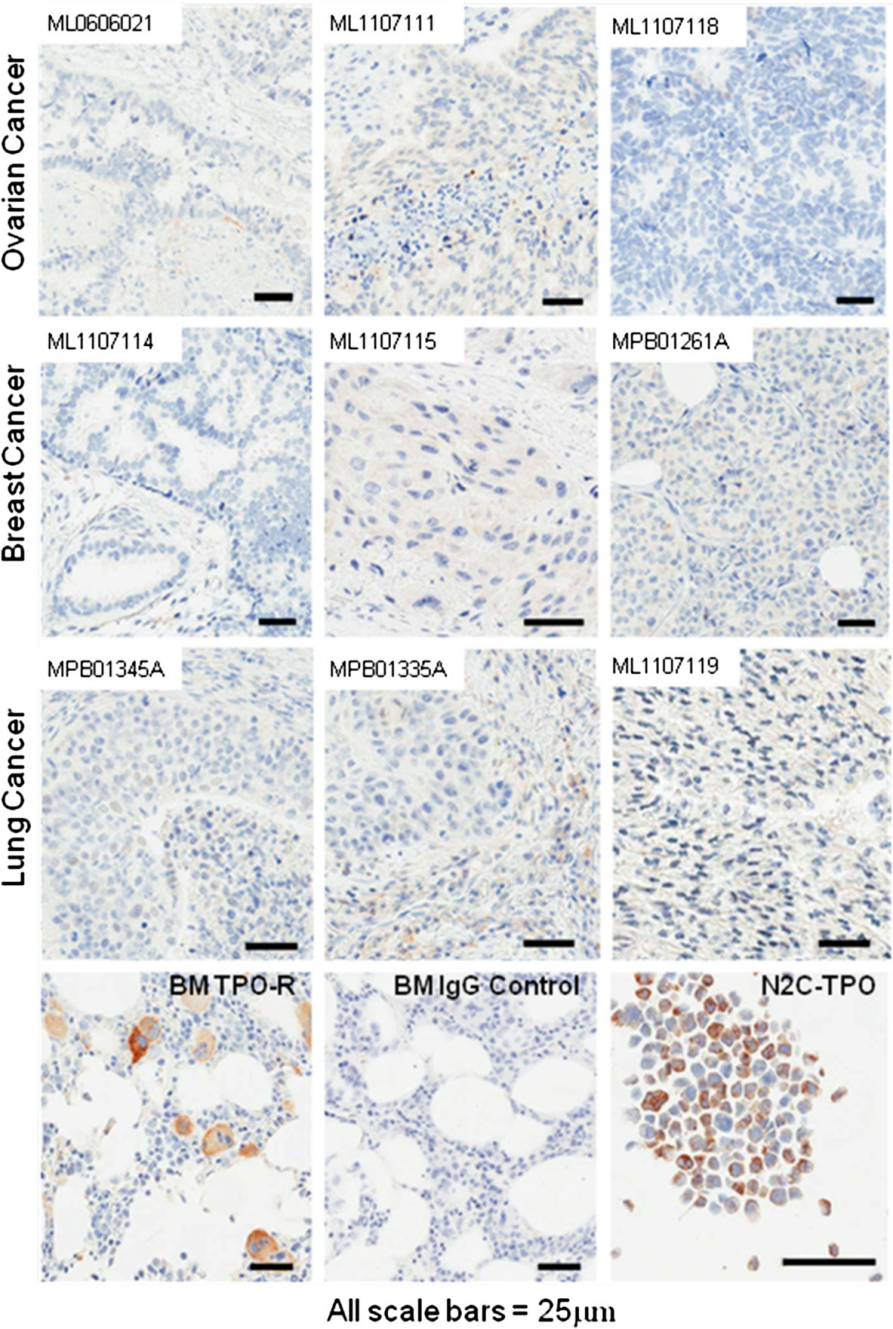
**Immunohistochemistry staining of breast, lung, and ovarian tumor samples for TPO-R.** Bottom panel: specific staining of megakaryocytes in normal bone marrow with anti-CD110 (TPO-R) versus the isotype control (IgG) and staining of positive control N2C-Tpo cell line.

Immunohistochemical analysis examining multiple fields of FFPE samples of breast, lung, and ovarian patient tissue stained with the anti-TPO-R antibody (CD110) demonstrated little or no specific signal. One breast tumor section showed a 1+ signal in one of infiltrating inflammatory cell, but no staining of tumor cells was noted. (Figure 4 and Additional file 2: Table S2). As expected, the positive controls of normal bone marrow megakaryocytes and the N2C-Tpo cell line (Figure 4 bottom panels) demonstrated robust specific staining with the antibody.

### Cell line proliferation assays

**Figure 5:**
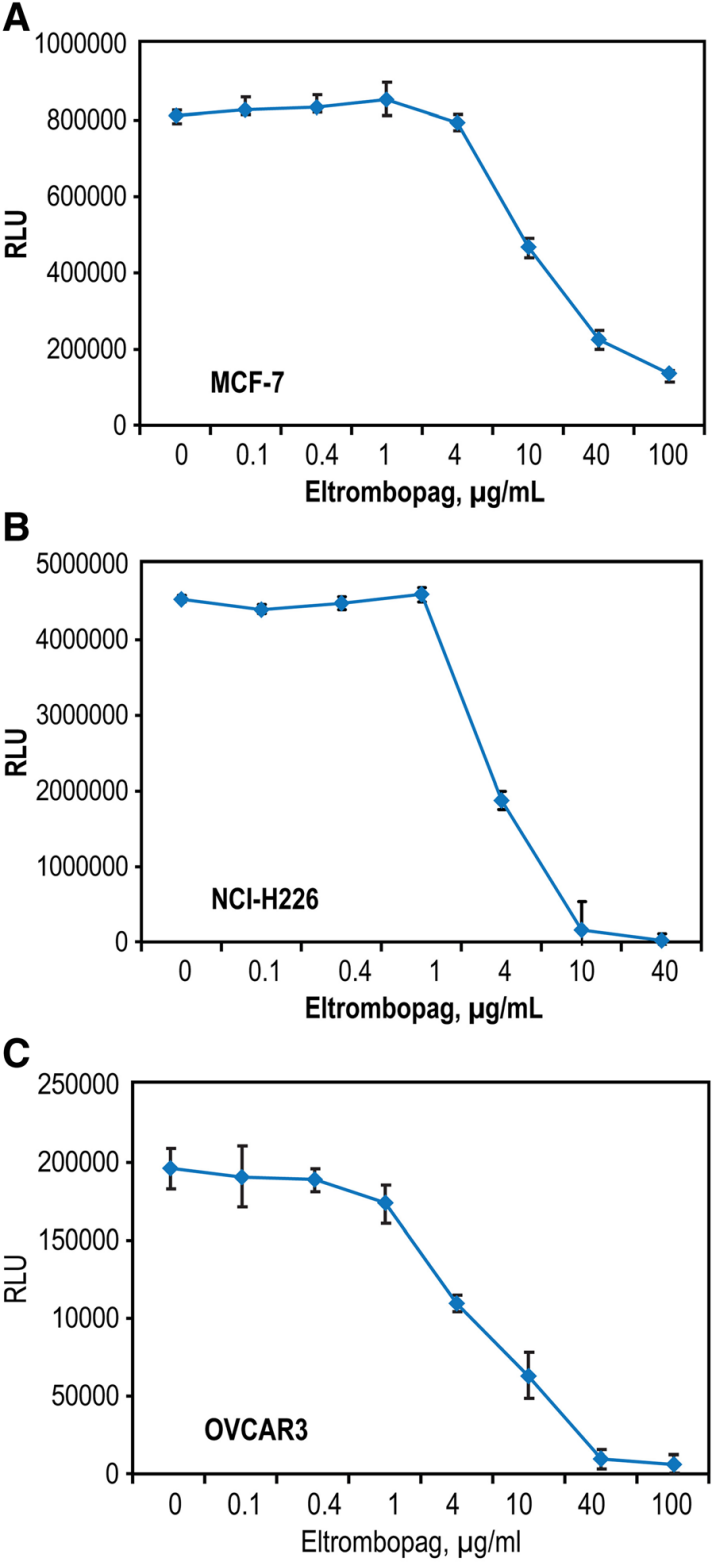
**Representative experiment demonstrating the effect of eltrombopag on proliferation of breast (MCF-7), lung (A549), and ovarian (OVCAR3) carcinoma cell lines.** Data are reported as relative luminescence units (RLU). (**A**). MCF-7, breast cell line. (**B**). NCI-H226 cells, lung squamous carcinoma cell line. (**C**). OVCAR3, ovarian carcinoma cell line.

Given that the expression of mRNA does not always reflect the expression of protein, several breast, lung, and ovarian cancer cell lines were chosen to assess the effects of eltrombopag on proliferation using the Cell Titer Glo assay. Three different breast cancer cell lines MCF-7, BT474, and HCC1937 were incubated with eltrombopag for 72 hours to determine whether this compound stimulated proliferation of breast cancer cells. The MCF-7 cell line data are shown as a representative experiment in Figure 5A. No increase in cell number was observed in the 3 breast cancer cell lines with eltrombopag (0.1-100 μg/mL); in fact, all of them showed a decrease in cell number at eltrombopag concentrations > 4 μg/mL. Recombinant TPO did not increase or decrease proliferation of any of these breast cancer cell lines (data not shown). Table 3 shows the IC_50_ of eltrombopag on these cell lines: 19.0 μg/mL for MCF-7; 9.6 μg/mL for BT474; and 10.7 μg/mL for HCC1937.

**Table 3:** **IC**_**50 **_**of proliferation of eltrombopag on breast, lung, and ovarian tumor cell lines**

**Cell line**	**Tumor type**	**IC**_**50**_**(μg/mL)**
MCF-7	Breast carcinoma	19.0
BT474	Breast carcinoma	9.6
HCC1937	Breast carcinoma	10.7
A549	Lung alveolar cell carcinoma	9.0
NCI-H226	Lung squamous cell carcinoma	3.7
NCI-H460	Large cell lung carcinoma	8.1
NCI-H510	Small cell lung carcinoma	10.3
OVCAR3	Ovarian adenocarcinoma	4.8
OVCAR4	Ovarian carcinoma	11.0
SKOV-3	Ovarian adenocarcinoma	49.7

Four lung carcinoma cell lines were studied to determine whether eltrombopag stimulated proliferation including 3 NSCLC (A549, from an alveolar cell carcinoma; NCI-H226, from a squamous cell carcinoma; and NCI-H460, from a large cell carcinoma) and a small cell lung carcinoma cell line, NCI-H510. There was no increase in cell number over 72 hours of treatment with eltrombopag at concentrations from 0.1 μg/mL to 40 μg/mL in any of the 4 lung carcinoma cell lines. The response of NCI-H226 to eltrombopag is shown in Figure 5B. All cell lines showed a decrease in cell number at higher concentrations of eltrombopag (>4 μg/mL). Recombinant TPO did not affect the proliferation, either positively or negatively, of any of these cell lines (data not shown). As shown in Table 3, the IC_50_ of eltrombopag on these cell lines was 9.0 μg/mL for A549; 3.7 μg/mL for NCI-H226; 8.1 μg/mL for NCI-H460; and 10.3 μg/mL for NCI-H510.

None of the ovarian carcinoma cell lines (OVCAR3, OVCAR4, and SKOV-3) demonstrated increased proliferation in response to 0.1 μg/mL to 100 μg/mL eltrombopag treatment over 72 hours. For all 3 cell lines, cell number decreased at eltrombopag concentrations > 4 μg/mL. A representative experiment demonstrating the response of OVCAR3 cells is shown in Figure 5C. Recombinant TPO had no positive or negative proliferative effect on these cell lines (data not shown). As shown in Table 3, the IC_50_ of eltrombopag on these cell lines was 4.8 μg/mL for OVCAR3, 11.0 μg/mL for OVCAR4, and 49.7 μg/mL for SKOV-3.

### Cell line TPO-R protein expression

**Figure 6:**
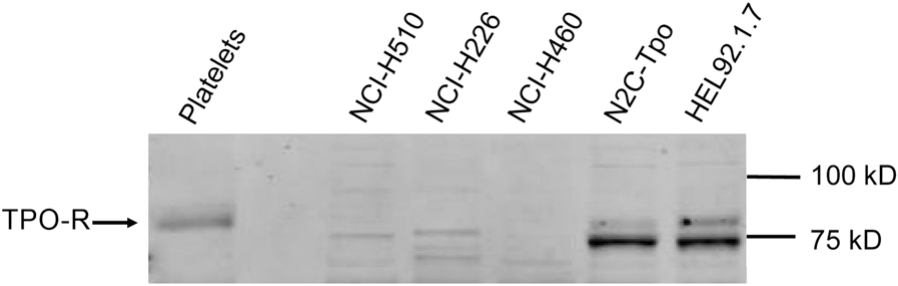
**Western blot of TPO-R protein expression in lung carcinoma cell lines**.

We had previously noted that NCI-H510, the cell line derived from small cell lung carcinoma, expressed high levels of *MPL* mRNA by qRT-PCR [31]. We sought to determine whether mRNA expression correlated with TPO-R protein expression using Western blots of cell lysates of lung cancer cell lines treated with eltrombopag. In NCI-H510 cell lysates, no band corresponding to TPO-R protein was detected. Human platelets and the megakaryocytic cell lines N2C-Tpo and HEL92.1.7 had high levels of *MPL* mRNA and TPO-R protein (Figure 6). NCI-H226 and NCI-H460 cell lines did not express detectable *MPL* mRNA by qRT-PCR, nor did they demonstrate TPO-R protein on Western blots. Western blots reveal a band corresponding to a protein smaller than TPO-R in NCI-510 and NCI-H226 cell lysates that is also present in N2C-Tpo and HEL92.1.7 lysates, but the identity of this band is unknown.

Thrombocytopenia is commonly observed during treatment with a number of chemotherapy regimens approved or in development for the treatment of breast, lung, and ovarian cancers [8,32-36]. Identification and characterization of novel, safe, and effective thrombopoietic agents to ameliorate thrombocytopenia remains an intense area of research [20]. Eltrombopag interacts with TPO-R, stimulates megakaryopoiesis, and promotes platelet production and maturation. Although *MPL* is expressed on megakaryocytes and megakaryocyte precursors [15-18], little quantitative data are available on *MPL* expression in other tissues.

In this study, *MPL* expression was compared with expression of other genes (*EPOR*, *ERBB2*, and *IGF1R*), which have been widely reported [37-42]. Our analyses showed that *MPL* was not commonly expressed at detectable levels in tumor samples as measured by qRT-PCR. Using microarray analyses, *MPL* expression was lower than other cell surface receptors. Even when protein is expressed, it may not be folded properly to allow ligand interactions. For example, although *MPL* is expressed on leukemic blasts from patients with AML [23], no proliferation in response to eltrombopag in bone marrow mononuclear cells from patients with AML or MDS has been observed [26]. Nor did eltrombopag stimulate proliferation in a variety of non-megakaryocytic leukemia and lymphoma cell lines; decreased proliferation was observed at physiologically achievable eltrombopag concentrations (≥ 4 μg/mL) [19]. The observed median Cmax for eltrombopag in patients with ITP is 11.4 μg/mL at the 75-mg dose [19], while in patients with chemotherapy-induced thrombocytopenia, the observed median Cmax at the 75-mg dose (based on sparse data) is approximately 9.90 μg/mL (unpublished data). Therefore, the dose at which tumor cell line growth declines is in a physiologically achievable range. Further studies are needed to determine whether eltrombopag affects tumor growth in vivo.

## Conclusions

Eltrombopag did not stimulate growth of breast, lung, or ovarian cancer cell lines at doses likely to exert action on megakaryocytes and megakaryocyte precursors. Tumor samples of these types had very little, or no, *MPL* mRNA or TPO-R protein expression as determined by qRT-PCR or IHC, respectively.

## Abbreviations

*ACTB*: β-actin; AEs: Adverse events; AML: Acute myeloid leukemia; Bp: Base pair; CT: Cycle time; FCS: Fetal calf serum; FFPE: Formalin-fixed parafin embedded; FISH: Fluorescence in situ hybridization; *GAPDH*: Glyceraldehyde-3-phosphate dehydrogenase; IHC: Immunohistochemistry; IMDM: Iscove’s Modified Dulbecco’s Medium; IRB: Institutional Review Board; ITP: Chronic immune thrombocytopenia; MDS: Myelodysplastic syndromes; NSCLC: Non-small cell lung cancer; *PPIA*: Cyclophilin A; qRT-PCR: Quantitative reverse transcription-polymerase chain reaction; rhTPO: Recombinant human TPO; RLU: Relative luminescence units; RMA: Robust microchip analysis; RT-PCR: Reverse transcription-polymerase chain reaction; TPO: Thrombopoietin; TPO-R: Thrombopoietin receptor.

## Competing interests

CLEM is employed by GlaxoSmithKline (GSK), holds stock, and receives research funding. KP is a former employee of GSK, held stock, and received research funding. JK is employed by GSK. DJF, LO, AMM, YL, YMK, and CM are employed by GSK and hold stock.

## Authors’ contributions

CLEM contributed to the conception and design of the experiments, analyzed and interpreted data, drafted and critically reviewed the manuscript. KP carried out gene profiling and cell proliferation assays. JK performed qRT-PCR and cell proliferation assays. DJF participated in IHC data acquisition, and analysis and interpretation. LO participated in data acquisition and manuscript revisions. AMM conducted research that contributed to results and helped in writing and editing the manuscript. YL participated in data acquisition and data analysis and interpretation. YMK participated in the interpretation of data and critical review of the manuscript. CM contributed to the conception and design of the experiments, interpreted the data, and drafted and critically reviewed the manuscript. All authors read and approved the final manuscript.

## Pre-publication history

The pre-publication history for this paper can be accessed here:

http://www.biomedcentral.com/1471-2407/12/405/prepub

## Supplementary Material

Additional file 1 **Table S1.** Sequences of primers and probes utilized in qRT-PCR analyses. qRT-PCR, quantitative reverse transcription-polymerase chain reaction. Click here for file

Additional file 2 **Table S2.** Pathologists review of immunohistochemistry slides. Click here for file
