# Supplementary material for: Low or undetectable TPO receptor expression in malignant tissue and cell lines derived from breast, lung, and ovarian tumors
Source: BMC Cancer. 2012 Sep 11;12:405. doi: 10.1186/1471-2407-12-405 (PMC3480928; doi:10.1186/1471-2407-12-405)
Supplement: Additional file 1 — Table S1. Sequences of primers and probes utilized in qRT-PCR analyses. qRT-PCR, quantitative reverse transcription-polymerase chain reaction. [file 1471-2407-12-405-S1.docx]

**SUPPLEMENTAL DATA**

**Supplemental Data Table 1.** **Sequences of primers and probes utilized in qRT-PCR analyses**

| **TPO-R** |  |
| --- | --- |
| **Forward** | **AGTGGAACCCAGCCTCCTTG** |
| **Reverse** | **CTGCAATCTTCGGTAGTCCATCTG** |
| **Probe** | **FAM-CAAGTCCTCAGAGGACTCCTTTGCCC-TAMRA** |
| **EPOR** |  |
| **Forward** | **GCTATGTGGCTTGCTCTTAGGACAC** |
| **Reverse** | **CCATCCCTGTTCCATAAGTCTTGAG** |
| **Probe** | **FAM-ATTGGATCCCTGATCATCTGCAGCCTG** |
| **ERBB2** |  |
| **Forward** | **AGCCCAGCCTTCGACAACC** |
| **Reverse** | **GTCCCTTTGAAGGTGCTGGG** |
| **Probe** | **FAM-ATTACTGGGACCAGGACCCACCAGAGC** |
| **IGF1R** |  |
| **Forward** | **GATTCAGATGGCCGGAGAGA** |
| **Reverse** | **TGTGGACGAACTTATTGGCG** |
| **Probe** | **FAM-TGCAGACGGCATGGCATACCTCA** |
| **GAPDH** |  |
| **Forward** | **CAA GGT CAT CCA TGA CAA CTT TG** |
| **Reverse** | **GGG CCA TCC ACA GTC TTC TG** |
| **Probe** | **FAM-ACC ACA GTC CAT GCC ATC ACT GCC A-TAMRA** |
| **Cyclophilin A** |  |
| **Forward** | **CAT CTG CAC TGC CAA GAC TGA** |
| **Reverse** | **TGC CTT CTT TCA CTT TGC CA** |
| **Probe** | **FAM-CAC CAC ATG CTT GCC ATC CAA CCA-TAMRA** |
| **β-actin** |  |
| **Forward** | **GAG CTA CGA GCT GCC TGA CG** |
| **Reverse** | **GAT GTT TCG TGG ATG CCA CAG GAC** |
| **Probe** | **FAM-CAT CAC CAT TGG CAA TGA GCG GTT CC-TAMRA** |

qRT-PCR, quantitative reverse transcription-polymerase chain reaction.
