# Supplementary figures and images for: Low or undetectable TPO receptor expression in malignant tissue and cell lines derived from breast, lung, and ovarian tumors
Source: BMC Cancer. 2012 Sep 11;12:405. doi: 10.1186/1471-2407-12-405 (PMC3480928; doi:10.1186/1471-2407-12-405)

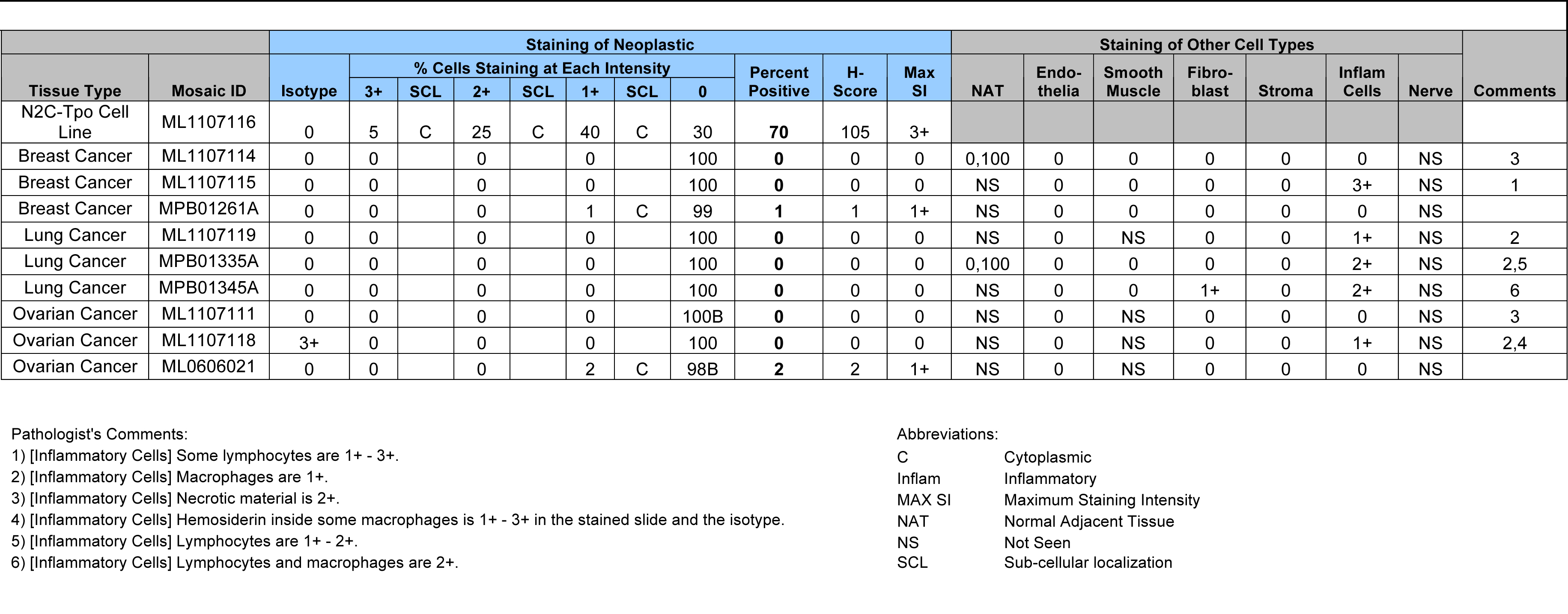

Supplement: Additional file 2 — Table S2. Pathologists review of immunohistochemistry slides. [file 1471-2407-12-405-S2.jpeg]
